# Optimization of Ultrasound-Assisted Extraction Condition for Phenolic Compounds, Antioxidant Activity, and Epigallocatechin Gallate in Lipid-Extracted Microalgae

**DOI:** 10.3390/molecules25030454

**Published:** 2020-01-21

**Authors:** Da Hye Gam, Song Yi Kim, Jin Woo Kim

**Affiliations:** 1 Department of Food Science, Sunmoon University, Natural Science 118, 70 Sunmoon-ro 221, Tangjeong-myeon, Asan-si, Chungnam 336-708, Korea; ank7895@naver.com (D.H.G.); kimjw1028@sunmoon.ac.kr (S.Y.K.); 2 FlexPro Biotechnology, Natural Science 128, 70 Sunmoon-ro 221, Tangjeong-myeon, Asan-si, Chungnam 336-708, Korea

**Keywords:** microalgae, phenolic compounds, antioxidant, Epigallocatachin gallate, response surface methodology, ultrasound-assisted extraction (UAE)

## Abstract

Lipid-extracted microalgae (LEM, *Tetraselmis* KCTC 12236BP), a solid waste by-product obtained from algal biodiesel production, is typically considered a rich source of antioxidant compounds, including phenolic compounds. The purpose of this study was to apply a statistically-based methodology to enhance the extraction of total phenolic compounds (TPCs) and antioxidant activity (AA) from LEM and to verify the production of epigallocatechin gallate (EGCG), a bioactive material, under optimum conditions. The optimal extractions of TPC and AA were explored by varying the key variables, including the extraction temperature, ethanol concentration, extraction time, and ultrasonic power, through statistical optimization. The optimal extraction conditions were identified through 27 runs following the central composite design. The regression analyses of TPC and AA showed good fit of the experimental data to the second-order polynomial models, with coefficient of determination (R^2^) values of 0.8769 and 0.8432, respectively. In the variation experiment, the maximum TPC and AA values of 9.8 mg GAE/g and 91.8% were obtained respectively with an extraction temperature of 74.4 °C, ethanol concentration of 55.4%, extraction time of 59.6 min, and ultrasonic power of 700 W. HPLC coupled with diode array detection was used to identify and quantify the phenolic compounds in the extracts, and EGCG (0.12 mg/g DM) was identified as a major peak in the analysis, demonstrating that high value-added material with a bioactive property can be produced from LEM. The results indicated that statistical optimization is applicable for optimizing the extraction of TPC and AA from LEM and provided a scientific basis for applying ultrasound-assisted extraction on an industrial scale by optimizing the conditions. LEM has a high TPC value, particularly with regard to EGCG, and excellent AA, considering it is highly used as a functional material for food, cosmetics, and medicine.

## 1. Introduction

Free radicals are generated during normal cellular function and are part of the natural physiological mechanism of all living cells. They are oxygen-containing molecules with an uneven number of electrons that are derived from normal metabolic processes, as well as external stresses such as exposure to radiation, ozone, cigarette smoke, air pollutants, and industrial chemicals. When large amounts of free radicals are produced in the human body, their accumulation and the consequent oxidation of cell components occur during a metabolic process called “oxidative stress” [1], which leads to a chain reaction that causes free radicals to play a significant pathological role in human diseases [2,3].

Antioxidants in certain plant foods can neutralize free radicals by donating electrons to delay or halt cellular damage through a free radical scavenging property [4]. Plant foods, such as fruits, vegetables, and grains, contain various beneficial components that protect the body from oxidative damage. For effectively controlling oxidative damage, the development of new antioxidants from natural resources is important, and various studies have been performed to determine methods to prevent the damage from free radicals. Currently, to reduce the negative effects of free radicals, the food industry has extensively been using synthetic antioxidants including butylated hydroxytoluene, butylated hydroxyanisole, and tert-butylhydroquinone. The synthetic antioxidants accepted as food additives are inexpensive and possess effective antioxidant abilities [5]. However, reportedly, excessive consumption of synthetic antioxidants can result in gene mutations and cause various disorders including cancer and liver disease [6,7]. In addition, currently available synthetic antioxidants exhibit negative aspects such as low solubility, unpleasant odors, high volatility, and consumers’ reluctance [8]. Therefore, in most countries, the use of synthetic antioxidants is strictly regulated, and long-term safety evaluation has become mandatory because of the health hazard. Moreover, concerns with regard to the use of synthetic antioxidants and preference for natural ingredients have led to their substitution with natural antioxidants, despite the latter being more expensive and less effective than the former [9].

Plant secondary metabolism produces numerous functional compounds that are essential for plant survival in their particular environments. Approximately 20% of the carbon fixed by photosynthesis is accumulated into the phenylpropanoid pathway, and over 100,000 secondary metabolites are produced in plants [10,11]. In nature, plants protect themselves against biotic and abiotic stresses primarily through their chemical and biological defense systems using secondary metabolites [12]. These compounds typically belong to one of three large chemical classes: Phenolics, terpenoids, and alkaloids. Among these classes, phenolics represent the main group of secondary metabolites and are one of the most common and predominant substances in the plant kingdom [13,14]. Phenolics exhibit various biological properties such as anticancer, antioxidant, antimicrobial, anti-hypertensive, and anti-inflammatory activities [15]. Therefore, in recent years, the production of phenolics from plants, including herbs, fruits, vegetables, and microalgae, has garnered increasing interest commercially, including the food and beverage, cosmetic, and pharmaceutical industries [16,17].

Plant biomass is the most abundant renewable resource on the planet. Among the numerous options of plant biomass, microalgae have gained considerable attention as an alternative to terrestrial plants (which are considered second-generation biomass) because of their high growth rate, efficient lipid production, and CO_2_ accumulation. Therefore, microalgae are considered a third-generation biomass for the production of biofuels such as biodiesel and bioethanol. In addition to being a source of algal biofuels, microalgae have been recognized for their potential as industrially feasible natural sources of phenolic compounds owing to the need to enhance the price competitiveness of microalgae biodiesel, as well as the high demand to develop natural antioxidants as alternatives to synthetic antioxidants [18,19]. Ultrasonic-assisted extraction (UAE) is widely known as a suitable extraction technique for extracting bioactive substances from plant biomass. UAE is based on the principle of acoustic cavitation which is capable of destructing plant cell walls, favoring solvent penetration, mass transfer, and the release of bioactive compounds [20,21]. Improved extraction efficiency by ultrasound has been attributed to the propagation of ultrasound pressure waves, resulting in a cavitation effect with compression and expansion of bubbles in liquid. The growth and collapse of bubbles generate macro-turbulence, high-velocity inter-particle collisions, and perturbation in micro-porous particles of the plant biomass. These result in surface disrupture, particle breakdown, and internal diffusion leading to acceleration of mass transfer from the solid to liquid phase [22,23].

The aim of this study was to determine an effective extraction method for bioactive materials, including phenolic compounds and antioxidants, from lipid-extracted microalgae (LEM; *Tetraselmis* KCTC 12236B). A four-variable, five-level central composite design (CCD) was used to simultaneously enhance total phenolic compounds (TPCs) and antioxidant activity (AA) based on the most suitable conditions of temperature, duration, solvent, and ultrasound-assisted extraction (UAE) power. The bioactive properties of LEM obtained through UAE were evaluated to confirm its potential for use as a food, cosmetic, and medical ingredient.

## 2. Results and Discussion

### 2.1. Model Fitting and Statistical Analysis

A response surface method—CCD with four variables and five levels—was applied to evaluate the influence of key UAE process variables, including temperature (X_1_: 50–90 °C), ethanol concentration (X_2_: 21.8–94.8% *v/v*), extraction time (X_3_: 15–75 min), and ultrasonic power (X_3_: 300–700 W), on the extraction of TPC and AA (Table 1).

The five-level four-factor CCD was carried out to optimize the UAE condition. The TPC and AA total acquired from 27 groups of experiments are listed in Table 2. Overall, 27 runs were conducted for optimizing the four variables in a random order to minimize the effect of unexpected variability in the responses (Table 2). TPC and AA yields ranged from 5.10 to 9.41 mg GAE/g and from 24.7% to 84.8%, respectively. The highest TPC and AA values were obtained at runs no. 16 and no. 14, respectively.

The five-level four-factor CCD was carried out to optimize the UAE of TPC and AA using RSM. TPC and AA obtained from 27 sets of experiments were used for the generation of a regression equation. According to previous experimental results, two second-order polynomial regression equations and their mathematical expressions based on the coded value were automatically generated by the design expert software. The response variable and two fitted coding equations of the model are shown in Table 3.

The actual TPC and AA values obtained in the experiment were analyzed by multiple regression models to fit polynomial equations. The analysis of variance (ANOVA) used to determine the best correlation between each response indicated that the contribution of the quadratic model was significant and fit the experimental data better than other models. Therefore, the quadratic model was employed to predict the TPC and AA based on the data from the 27 experimental runs. The significance of the models, constant terms, the linear terms, the interaction terms, and the square terms were determined by analysis of variance (ANOVA) and the results are shown in Table 4.

The CCD generated two regression equations that demonstrated the empirical relationship between the response variables and process variables. Coefficients of the quadratic model were validated using ANOVA. The estimated regression coefficients and their sum of squares, *f*-values, and *p-*values are listed. The significance of fitting was validated using the coefficient of determination (R^2^), which is defined as the ratio of the explained variation to the total variation in response and is used to express the quality of a regression model [24]. The TPC and AA values were R^2^ = 0.8769 and R^2^ = 0.8432, respectively, demonstrating a good agreement between the experimental and predicted values. The significance of each coefficient was determined using the *f*-test and *p*-value. According to the results, the model was highly significant when the computed *f*-value was greater than the tabulated *f*-value and the probability value was low (*p* < 0.05) [25]. ANOVA showed that the model’s *f*-values were 9.09 and 5.28 for the TPC and AA, respectively, with probability (*p* < 0.001), implying there was only a 0.1% chance that this large *f*-value occurred because of noise and the model was remarkably significant and can be used for subsequent optimization designs.

**Figure 1:**
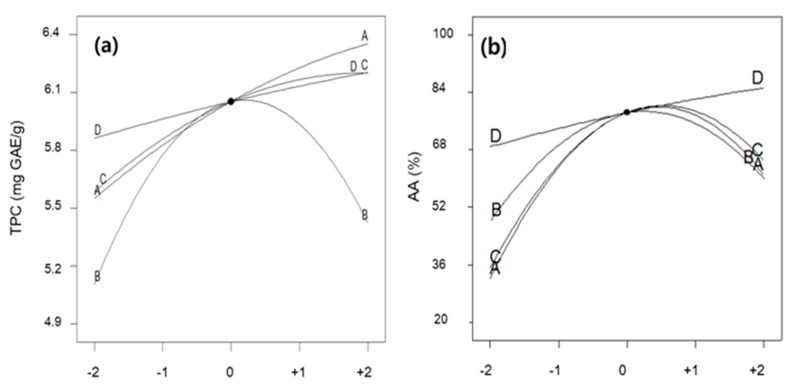
Effects of independent variables on total phenolic compounds. (**a**) Extraction and antioxidant activity, (**b**) one variable was changed with fixed levels of the other factors. Each level of variables was expressed as the code values. A: Extraction temperature; B: Ethanol concentration; C: Extraction time; D: Ultrasonic power.

As determined by ANOVA, the quadratic model generated by the 27 experimental runs was sufficient for the prediction of the TPC and AA values. Based on the results of ANOVA, all variables, except ultrasonic power (X_4_), temperature (X_1_), ethanol concentration (X_2_), and extraction time (X_3_), showed significant quadratic effects on TPC and AA (*p* < 0.05). Therefore, as shown in the perturbation curves in Figure 1a,b, when the levels of the three variables (X_1_, X_2_, and X_3_) varied from the coded levels between −2.0 and 2.0, TPC and AA exhibited an upward convex curve over the process variables, except for ultrasonic power (X_4_). According to the ANOVA table, extraction temperature and time exhibited the most significant linear effects among the tested variables (*p* < 0.001), as shown in the perturbation curves.

### 2.2. Optimization of UAE Condition

Perturbation curve shows the comparison between all variables at a particular point and helps to compare the effect of all independent variables in the design space. The perturbation curves for the responses including TPC and AA are shown in Figure 1. A steep slope or curvature of variable shows that the response is sensitive to that variable and a relatively linear slope shows insensitivity to change in that particular variable. The yields of TPC and AA were plotted by changing one variable over its range while the other variables were held constant. TPC yield increment was observed as the temperature, time, and power were increased while the TPC yield decreases as the ethanol concentration was increased after the certain concentration. For AA level, by comparing the slope patterns of TPC, it was found that only power has a linear effect compared to other variables. AA levels tended to increase sharply with the increase of temperature, time, and concentration. As temperature, time, and concentration have a stronger effect compared to power, excessive extraction temperature, time, and concentration might not be effective for the extraction. This means that exposure to high temperatures for a long period of time may lead to product reductions because of the oxidation and polymerization process.

**Figure 2:**
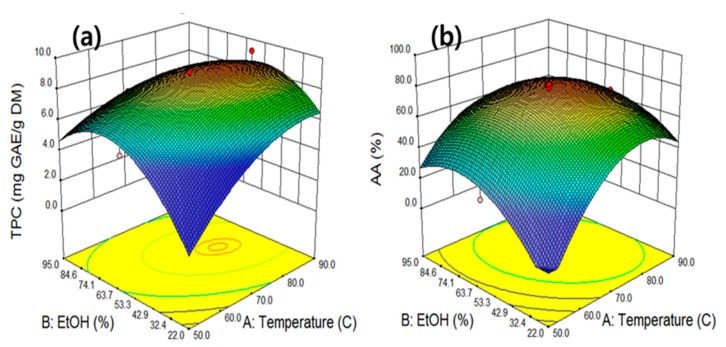
The interactive effects of the extraction temperature and ethanol concentration on total phenolic compounds (**a**) and antioxidant activity (**b**) at fixed levels of extraction time 45 min and ultrasonic power 500 W.

The interactive effect of the independent variables on the response was to generate three-dimensional response surface curves, which were conducted by varying two variables fixing the other two variables at center points. An appropriate choice of solvent concentration is essential for achieving an effective extraction. Ethanol and water are widely used in food industries because they are safer to handle compared with other organic solvents and are approved for use as solvents for food processing and human consumption. The interactive effect of extraction temperature and ethanol concentration at fixed levels of extraction time and ultrasonic power was investigated. In Figure 2a,b, the three-dimensional response surface curve shows that when the levels of extraction temperature and ethanol concentration were less than 79.3 °C and 56.6%, respectively, the TPC extraction increased along with these variables.

When the extraction temperature and ethanol concentration exceeded their optimum points, the extraction of TPC decreased with the increase of the two variables. The highest TPC concentration of 9.1 mg GAE/g was obtained at an extraction temperature of 78.9 °C and ethanol concentration of 56.6%. Figure 2b depicts the effects of temperature (X_1_) compared with ethanol concentration (X_2_) at a constant extraction time of 45 min and ultrasonic power of 500 W. The surface curve predicts that the maximum AA was achieved at an extraction temperature of 74.3 °C and ethanol concentration of 60.5%. The surface curve implies that upon increasing the extraction temperature to >73.7 °C, the AA level decreased. This might be explained by the extraction of antioxidant material having reached equilibrium with the temperature increase near 73.7 °C; degradation of the antioxidant material may have occurred with subsequent temperature increases [26]. Water is the most polar solvent among extraction solvents. However, the effect of extracting antioxidant materials, such as phenolic compounds, using water is not inefficient because of the higher viscosity of water compared with other solvents. Since the extraction efficiency of antioxidant material mainly depends on the polarity and viscosity of the solvents used, a single solvent using water may be ineffective. Therefore, the combination of water and ethanol was observed to be more effective in extracting phenolic compounds than a single solvent of water or ethanol [27].

**Figure 3:**
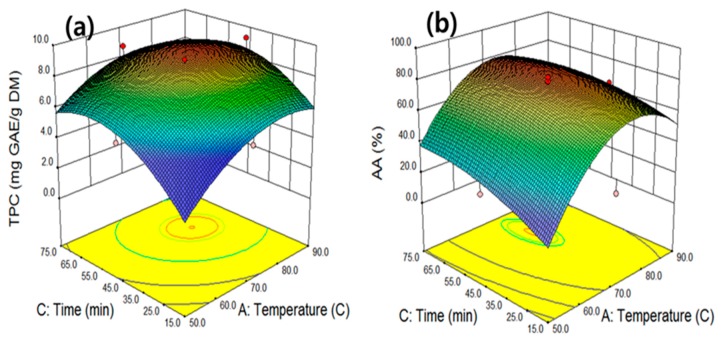
The interactive effects of the extraction temperature and time on total phenolic compounds (**a**) and antioxidant activity (**b**) at fixed levels of ethanol 58.3% and ultrasonic power 500 W.

As extraction temperature and time were determined to be the primary variables affecting the extraction of TPC and AA, experiments were conducted at different extraction temperatures and times with fixed levels of ethanol concentration and ultrasonic power (Figure 3a,b). The extraction of TPC increased with the increase in temperature and time up to 77.7 °C and 53.4 min, respectively. However, the extraction of TPC slightly decreased after these maximum points, implying that the further increases of extraction temperature and time were not advantageous for the extraction because of the degradation of the produced TPC. Figure 3b displays a response surface curve demonstrating the effect of extraction temperature and time on AA when the ethanol concentration and power were maintained at the central values of 58.3% and 500 W, respectively. Under this condition, the maximum AA obtained at 73.7 °C and 51.8 min was 81.4%. Increased temperature and extraction time led to the increase of TPC and AA because higher temperatures increase phenolic component solubility and reduce solvent viscosity, resulting in an increase of mass transfer up to a certain temperature and time. However, a continuous increase in the temperature and time resulted in the degradation of the extracted phenolic compounds from exhibiting AAs after reaching the equilibrium, resulting in a decrease in the TPC and AA levels [28].

**Figure 4:**
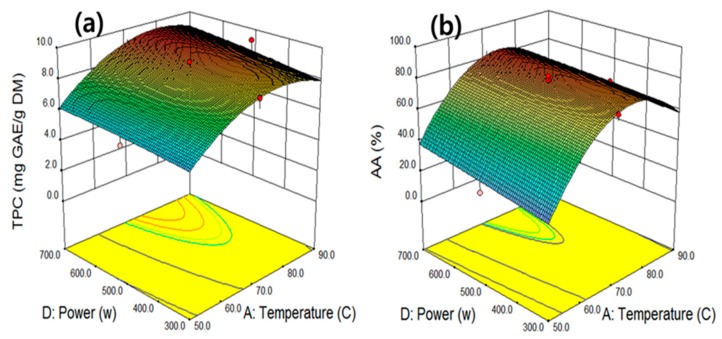
The interactive effect of temperature and ultrasonic power on total phenolic compounds (**a**) and antioxidant activity (**b**) at fixed levels of extraction time 45 min and ethanol concentration 58.3%.

The interactive effect of temperature and ultrasonic power was evaluated at fixed extraction time (45 min) and ethanol concentration (58.3%) levels (Figure 4a). Under these conditions, the maximum TPC value of 9.7 mg GAE/g was obtained at 80.2 °C and 700 W. TPC increased when the extraction temperature increased to 74.3 °C but exhibited a decreasing trend above the optimum temperature level. Unlike the other variables, the increase in TPC was proportional to the increase in ultrasonic power. The increase of ultrasonic power accelerates the destruction of cell walls, thereby promoting the extraction of the TPC into the extraction solvent. The surface curve in Figure 4b shows the interactive effect of extraction temperature (X_1_) and ultrasonic power (X_4_) at fixed levels of other variables. The surface curve shows that the maximum AA value occurred near the extraction temperature of 70 °C at all ranges of ultrasonic power. The maximum AA value (86.0%) was obtained under the UAE conditions of 73.3 °C and 698.6 W. Similar trends in phenolic compounds extraction were observed when the AA increased proportionally with increasing power, suggesting that the higher ultrasonic power caused the creation and collapse of bubbles, resulting in material swelling, solvent uptake, and pore enlargement in the materials, as well as in sufficient time for cavitation bubbles to extract TPC and antioxidants [29].

**Figure 5:**
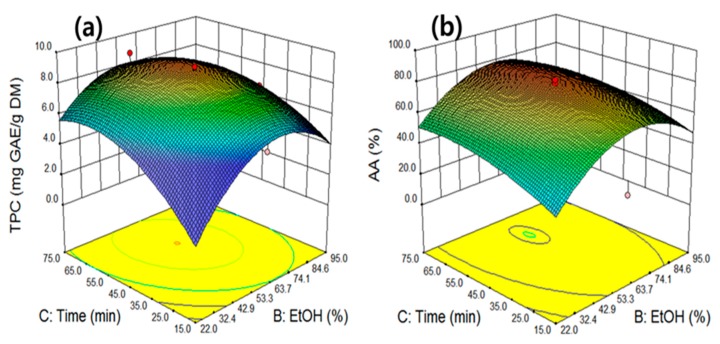
The interactive effect of ethanol concentration and time on total phenolic compounds (**a**) and antioxidant activity (**b**) at constant temperature 70 °C and power 500 W.

The response surface curve as a function of ethanol concentration (X_2_) versus time (X_3_) at a constant temperature of 70 °C and a power of 500 W is presented in Figure 5a. As reported in the ANOVA table, extraction time (*p* = 0.0005) showed a strong linearity because of the large influence of the linear term compared with ethanol concentration (*p* = 0.1391). The surface response curve reached the highest TPC value (9.0 mg GAE/g) when the ethanol concentration was increased, with extraction times of 58.9% and 54.1 min, respectively, and tended to decrease thereafter. Figure 5b illustrates the interactive effect of ethanol concentration (X_2_) versus time (X_3_) on the AA value; the response curve results were similar to those of TPC, demonstrating that the two variations exerted significant quadratic effects on AA. Therefore, AA significantly increased when ethanol concentration and time increased up to 62.6% and 52.8 min, respectively, and then decreased thereafter. When the extraction time increased, the probability of phenolic compounds making contact with the solvent increased as well, which led to a higher extraction efficiency. These extraction patterns indicated similar results as observed in the TPC value of the perennial herb *Curcuma zedoaria* [30], wherein the TPC value increased as the ethanol concentration and extraction time increased, until a maximum amount of TPC was obtained. Subsequently, the amount rapidly declined, as the rate of TPC decomposition increased faster than the rate of production. Although longer extraction times increased the TPC and AA values, along with the solid–liquid interaction, operation in optimal conditions remains important because of the risk of antioxidant compound degradation and increased operating costs. Plant cell walls contain chemical compounds with varying solubility for solvents with different polarities. Changes in the ratio of water to ethanol result in a change in solvent polarity and enhance the extraction of materials with a specific polarity. Here, the polarity of the ethanol–water mixture continuously decreased with the addition of ethanol to water. Adding ethanol to water further decreased the polarity of the solvent mixture, rendering it suitable for the extraction of nonpolar mixtures in plant cell walls.

**Figure 6:**
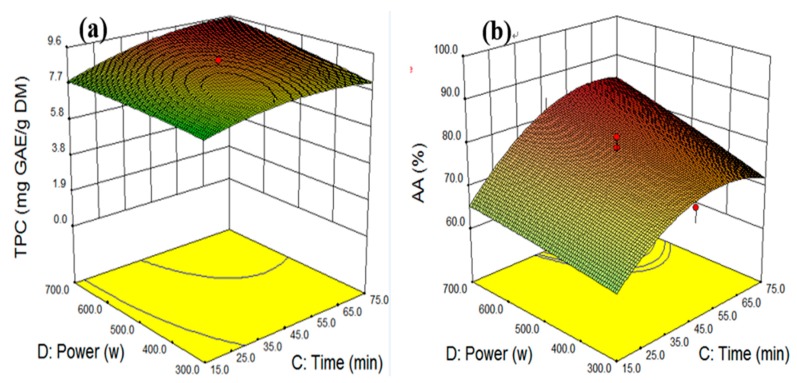
The interactive effect of extraction time and ultrasonic power on total phenolic compounds (**a**) and antioxidant activity (**b**) at constant levels of temperature 70 °C and ethanol concentration 58.3%.

Figure 6a illustrates the surface curve between extraction time and ultrasonic power at constant extraction temperature (70 °C) and ethanol concentration (58.3%) levels. The curve shows that the value of TPC obtained primarily depended on the linear term of ultrasonic power and quadratic term of extraction time. The highest level of TPC was 10.0 mg GAE/g when the extraction time and ethanol concentration were 62.5 min and 700 W, respectively. The TPC on the surface response curve began to level off at 62.8 min, indicating that the optimum extraction time was required to achieve the maximum TPC. The extended extraction time caused a breakdown of phenolic compounds. In Figure 6b, when the response surface curve was developed for the extraction time interaction with ultrasonic power, the maximum AA value was achieved under an extraction time of 57.6 min and ultrasonic power of 700 W. As the previous TPC experiments demonstrated, the extraction times showed a strong quadratic effect, with AA yield increasing to a peak of 90.2% before decreasing. An increase in the extraction time and power enhanced cell membrane breakdown, which increased the permeability of the solvent into the cell wall [31]. The highest AA activity was achieved upon increasing the ultrasonic power with water and ethanol. Therefore, the UAE extraction time of 52.5 min was selected as the optimum condition because prolonged extraction times used more energy and decomposed extracted bioactive compounds.

### 2.3. HPLC-DAD Analysis

**Figure 7:**
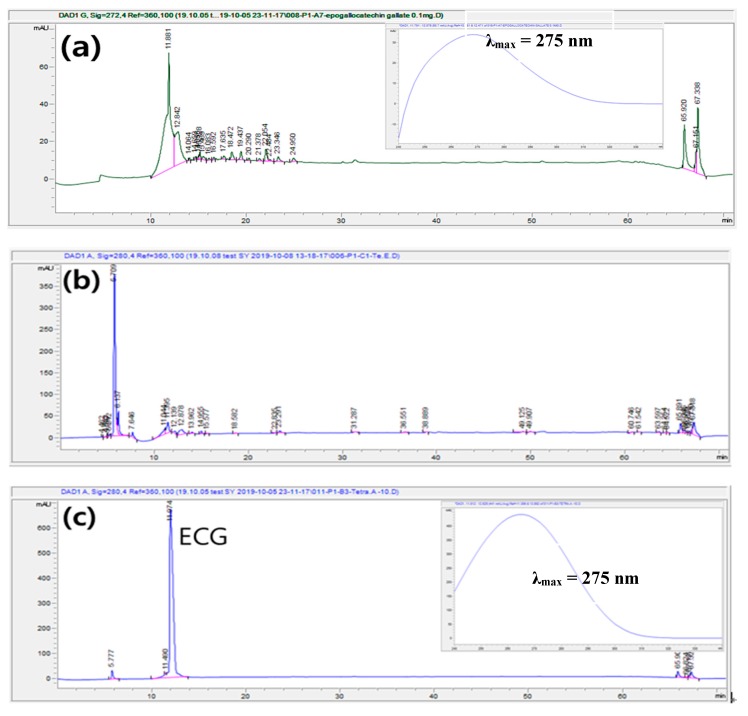
HPLC (high-performance liquid chromatography) chromatograms of epigallocatechin gallate (EGCG) standard (**a**), phenolic compounds from lipid extracted microalgae (LEM) extracted by water, (**b**) and by ethanol under optimized UAE condition (**c**). (**a**) Chromatogram of EGCG standard solution in water (0.1 mg/mL), (**b**) chromatogram of EGCG from LEM extracted by UAE using water (74.4 °C, 59.6 min, water), (**c**) chromatogram of EGCG from LEM extracted by UAE using ethanol (74.4 °C, 59.6 min, 55.4% ethanol).

**Figure 8:**
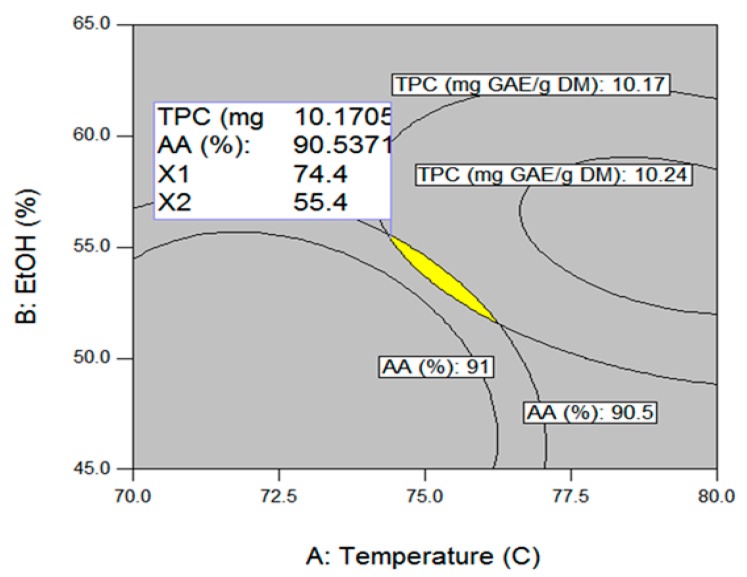
Optimization of extraction of total phenolic compounds and antioxidant activity based on the superimposing method. The interactive effect of extraction time and ultrasonic power on total phenolic compounds and antioxidant activity at constant levels of extraction time of 59.6 min, and ultrasonic power of 700 W.

In a previous experiment, the phenolic compounds content and antioxidant effects of LEM extracted via ultrasound were optimized; however, further research to validate the active ingredient was required. Therefore, phenolic compounds from LEM were identified using HPLC, which compared retention times (RTs) with those of standards, and using an extensive DAD spectral analysis between 240 and 340 nm. The HPLC chromatogram of water extracted from LEM showed one major peak at the RT of 5.71 min; however, no matching peak was identified among the standards in terms of RT and DAD spectrum. An HPLC-DAD analysis of ethanol extraction with the optimum UAE conditions revealed the presence of one major peak that showed good chromatography separation and resolution. The RT of the peak was 11.97 min. When the separated peak was identified according to RT and spectrum obtained from DAD in comparison with standard compounds (Figure 7), it was quantified as epigallocatechin gallate (EGCG), a type of catechin (also known as epigallocatechin-3-gallate) consisting of epigallocatechin and gallic acid. Figure 8 shows the chemical structure of EGCG (C_22_H_18_O_11_) with a maximum absorbance of 275 nm and was shown to have maximum absorbance of the same range as the results of previous studies [32].

It is considered that EGCG is the primary extract in the extraction of phenolic compounds from LEM through UAE because most phenolic compounds are removed during the preceding lipid extraction process. EGCG is a flavonoid found in several plants—particularly tea—that is effective in preventing cancer, heart disease, diabetes, and obesity. The ethanol extraction through UAE of LEM contained 0.12 mg EGCG/g dry LEM. These results demonstrated that LEM is a good source of phenolic compounds with strong antioxidant power and can be used in EGCG production with various physiological activation functions.

### 2.4. Validation of Model

As mentioned previously, the purpose of this study was to determine the UAE conditions that provide the maximum levels of TPC and AA. The results of the simultaneous optimization using the desirability function approach and each optimum condition were superimposed to predict the optimum points to satisfy the maximization of TPC and AA. Under the optimum conditions of temperature 74.4 °C, ethanol 55.4%, extraction time 59.6 min, and ultrasonic power 700 W, the TPC and AA values were 10.2 mg GAE/g and 90.5%, respectively (Figure 8).

The optimum extraction conditions were evaluated through a validation experiment with three repeated verification experiments. The obtained TPC and AA values were 9.8 mg GAE/g and 91.8%, respectively. These results confirmed that the experimental values were in agreement with the predicted values, suggesting that the model parameters obtained by CCD were accurate and reliable (Table 5).

## 3. Materials and Methods

### 3.1. Marine Microalgae

A strain of *Tetraselmis* (KCTC 12236BP) was isolated from Young-Heung island on the west coast of Korea and cultivated in an artificial seawater F/2 medium at Inha University (Marine Bioenergy Research Center, Incheon, Korea). The artificial seawater had the following composition: NaCl 24.7; KCl 0.67; CaCl_2_·2H_2_O 1.36; MgCl_2_·6H_2_O 4.66; MgSO_4_·7H_2_O 6.29; NaHCO_3_ 0.18 g/L. *Tetraselmis* were cultivated in aerated photo bioreactors (5 L, Korea Biotech., Incheon, Korea) under the following conditions: Aeration rate of 0.25 v.v.m. with 5% CO_2_, continuous light supply with intensity of 120 μmol m^−2^·s^−1^, temperature of 25 °C. The harvested *Tetraselmis* cells were washed twice with deionized water, and moisture was evaporated from the cells by oven drying at 60 °C for 8 h.

### 3.2. Lipid Extraction

Lipid was extracted using two organic solvents: Hexane (96%, Junsei, Tokyo, Japan) and methanol (99.6%, Junsei, Tokyo, Japan). The solid loading of LEM was 10% (*w/w*), and the mixture was stirred at 250 rpm for 6 h at room temperature for lipid extraction. Then, the mixture was separated into organic solvent and LEM by 6000 rpm centrifugation for 15 min (Ultra 5.0, Hanil Sci., Gimpo, Korea). Once the organic solvent was separated by decanting, washed with Milli-Q water for further analysis, and the residual solvent in LEM was removed using a vacuum evaporator (Eyela, FDU-1200, Tokyo, Japan). Then, the cells were ground into a powder with a Wiley mill (Model 4, Thomas Scientific, NJ, USA) and stored at −20 °C until they underwent UAE experiment.

### 3.3. Chemicals

Ethanol was purchased from Samchun chemical (96 *v/v*%, Seoul, Korea). Folin–Ciocalteu reagent, gallic acid (97%), and quercetin were purchased from Merck (Kenilworth, NJ, USA). 2,2-Diphenyl-1-picrylhydrazyl (DPPH) was purchased from Sigma-Aldrich (St. Louis, MO, USA). All other chemicals used in this experiment were of analytical grade and purchased from Sigma-Aldrich. All the stock solutions were prepared by purified deionized water using a Milli-Q purification system (Millipore, Burlington, USA).

### 3.4. Ultrasound-Assisted Extraction (UAE)

Phenolic compounds were extracted through UAE using a binary solvent system using water and ethanol. An ultrasonic bath (U02-56-249, Lk Lab., Namyangju, Korea) was used for the extraction. The temperature was controlled using a resistance thermometer and ultrasonic power was digitally controlled between 0 and 700 W with 40 Hz ultrasonic wave frequency. The range for the CCD experiment was based on the results obtained from the one-factor-at-a-time (OFAT) experiment conducted in our laboratory. As shown in Table 1, statistically-based optimization was used to evaluate the effect of extraction temperature (X_1_; 50–90 °C), ethanol concentration (X_2_; 21.8–94.8 *v/v*%), extraction time (X_3_; 15–75 min), and frequency (X_4_; 300–700 W). Sample of 0.5 g dried microalgae was placed into air-tight tube (25 mL), soaked with 10 mL of binary solvent at the given, and then placed in an ultrasound bath at 40 kHz for a certain time, a constant temperature, and ultrasonic power. At the selected time, a sample was taken and cooled at 5 °C water and centrifuged at 2500 g for 15 min. Extracts were separated from remaining solids by decanting and filtered with a disk-filter system with a pore size of 0.45 μm (26 mm, Supelco, Bellefonte, USA). Each 100 μL of extract was taken from the filtered extracts and diluted 10–100 times prior to the determination of TPC and AA analysis.

### 3.5. Total Phenoicl Content (TPC)

TPC was measured using the modified Folin–Ciocalteu method. Gallic acid was used as a standard chemical and calibration curves were plotted using five different gallic acid concentrations between 0 and 500 mg/L. The amount of extracted phenolic compounds was expressed as TPC based on the gallic acid equivalent to the initial amount of dried microalgae (mg GAE/g). In a 2.0 mL Eppendorf tube, 0.78 mL of distilled water, 0.02 mL of extract appropriately diluted, and 0.05 mL of Folin–Ciocalteu solution (Fisher Scientific Chem., Waltham, USA) were added and vortexed. After exactly 1 min of reaction, 0.15 mL of aqueous sodium carbonate of 20% (Na_2_CO_3_, 99.9%, Samchun Chemical, Nonhyun, Korea). The mixture was vortexed and allowed to react at room temperature in the dark for 60 min. The absorbance was measured at 765 nm by a UV–vis spectrophotometer (UV-1280, Shimadzu, Tokyo, Japan). Then, TPC was calculated from a calibration curve using the gallic acid (99%, Sigma-Aldrich, St. Louis, USA) as a standard and expressed as milligrams of gallic acid equivalents (GAE) per gram of dried matter. The equation of the gallic acid calibration curve was Y = 1.292·X−0.028 (where X was concentration of gallic acid equivalents (GAE) expressed as milligrams GAE per gram of dried extract and Y was measured absorbance, R^2^ = 0.9950)

### 3.6. Antioxidant Activity (AA) Assays

In order to evaluate AA, DPPH radical scavenging activity was measured as previously described [33]. Stock solution was prepared by mixing 75 mg of DPPH in 1 L of methanol overnight. A 0.5 mL aliquot of sample extract was mixed with 0.10 mL of DPPH solution. For each extract, a blank of 1.0 mL of methanol was used without DPPH reagent DPPH (2,2-diphenyl-1-picrylhydrazyl, Sigma-Aldrich, St. Louis, USA). The mixtures were vortexed and left standing for 30 min at 25 °C in the dark and the absorbance of each was measured at 520 nm. A lower absorbance of the reaction mixture indicated higher free radical scavenging activity. The ability to scavenge the DPPH radical was calculated using the following equation:

(1) $$\mathrm{Free}\;\mathrm{radical}\;\mathrm{scavenging}\;\mathrm{ability}\;\left(\mathrm{DPPH},\;\%\right)=\left[\frac{\left(A\;\mathrm{control}-A\;\mathrm{sample}\right)}{A\;\mathrm{control}}\right]\;\times 100$$

where A sample is the absorbance of the test extracts (containing DPPH solution) and A control is the absorbance of DPPH solution.

### 3.7. Experimental Design

A central composite design (CCD) with four variables with five levels (−2, −1, 0, +1, +2) was employed to determine the optimal UAE conditions for the maximization of TPC and AA, simultaneously. The design expert software (version 8.0, Stat-Ease Inc., Minneapolis, MN, USA) was applied and the four independent variables selected for UAE were extraction temperature (X_1_), ethanol concentration (X_2_), extraction time (X_3_), and power of ultrasonic power (X_4_). Table 1 shows the coded and actual values of the experimental variables. The complete design was performed randomly and consisted of 27 experimental runs including triplicated central point and axial points at distance ±2 from the central point (nine runs). Prior to RSM design, the single factors influencing the UAE with one-factor-at-a-time (OFAT) approach in our laboratory to determine the range of the CCD. Based on the OFAT experiments, the range of each level was determined as following, extraction temperature (50–90 °C), ethanol extraction concentration (21.8–94.8%), extraction time (15–75 min), ethanol extraction concentration (50% to 70%), and ultrasonic power (300–700 W). The experimental data from CCD were analyzed by multiple regressions to fit the following quadratic polynomial model as follows [34]:

(2) $$y=\beta_{0}+\;\overset{4}{\underset{i=1}{\sum }}\beta_{0}X_{i}\;+\;\overset{4}{\underset{i=1}{\sum }}\beta_{ii}X_{i}^{2}\;+\;\overset{4}{\underset{i=1}{\sum }}\overset{4}{\underset{j=1}{\sum }}\beta_{ii}X_{i}X_{j}$$

where *y* is the process response, *X_i_* is the coded independent variables, *β*_0_ is the intercept, and *β_i_*, *β_ii_*, and *β_ij_* are the linear, quadratic, and interaction regression coefficients, respectively.

Statistical analysis was performed using design expert and fitted to a quadratic polynomial model containing the coefficients of linear, quadratic, and interaction terms. An analysis of variance (ANOVA) was used to evaluate the statistical significance of the model [26]. ANOVA with 95% confidence level was carried out to analyze the significance of the model and equation terms. The sum of squares, f-, and *p*-values were used as the statistical parameters. The data achieved were subjected to a regression analysis using least square methodology to generate the equation that provided the response values as a function of the independent variables. The optimum UAE condition for the maximization of TPC and AA simultaneously were obtained by introducing the regression equation and a response surface curve was obtained using the fitted model by changing two variables with fixing the two variables simultaneously.

### 3.8. HPLC Analysis

Extracts were filtered prior to the analysis by 0.22 μm PVDF syringe filter (PVDF2025A, Hyundai micro, Seoul, Korea) and then analyzed by HPLC-Diode array detector (DAD) using an Agilent 1260 liquid chromatograph (Agilent technologies, Santa Clara, CA, USA) with quaternary pump and auto sampler. Separation was performed using Symmetry C18 column (250 × 4.6 mm, 5 μm, Agilent Zorbax SB-C18, Santa Clara, USA) at 25 °C. The mobile phase was composed of solvent A (99.9% *v/v*, acetonitrile, Sigma-Aldrich, St. Louis, USA) and solvent B (0.1% *v/v*, acetic acid, Dajung, Namyangju). The system gradient was run with the following gradient elution program (0 min, 0% A/100% B; 5 min, 15% A/85% B; 50 min, 50% A/50% B, 60 min, 100% A/0% B, 70 min, 0% A/100% B). The flow rate was kept constant throughout the analysis at 0.3 mL/min and the injection volume was 20 µL. The DAD detector was scanned for 240–340 nm for monitoring of the different groups of phenolic compounds. Phenolic standards, ascorbic acid, gallic acid, epigallocatechin gallate, chlorogenic acid, chatechin, caffeic acid, vanillic acid, syringic acid, coumaric acid, vanillin, sinapic acid, ferulic acid, benzoic acid, quercetine, and eugenol were purchased from Sigma-Aldrich (St. Louis, USA). Identification of phenolic compounds was performed by comparing their retention time and spectra with those of external standards.

## 4. Conclusions

Ultrasound is an emerging extraction process that is being thoroughly investigated for food applications. UAE was employed in this study, and extraction conditions were optimized to enhance TPC and AA extraction. The second-order regression model generated for the prediction of TPC and AA and the values predicted using the regression model matched well with the experimental values. A five-level four-factor CCD was applied to explore the linear, cross, and quadratic effects by using RSM of the four key parameters on TPC and AA. The optimal conditions of temperature 74.4 °C, ethanol 55.4%, time 59.6 min, and power 700 W yielded TPC and AA values of 10.2 mg GAE/g and 90.5%, respectively. Based on triplicated verification experiments, it was found that the experimental results were agreed well with the corresponding predicted values of 9.8 mg GAE/g and 91.8%, respectively. The following HPLC results confirmed high levels of TPC and AA via UAE of LEM, which was determined to be an effect resulting from EGCG, the main component of the extract. This is the first paper to investigate the oxidative capacity of phenolic compounds extracted from LEM and identify that EGCG is a major phenolic compound contributing to the antioxidant effect of LEM.

In the extraction of polyphenols including EGCG, conventional techniques use organic solvents that are not eco-friendly and have the disadvantage of destroying polyphenols at high temperature, oxidation, and long extraction time, so it is necessary to develop extraction methods to overcome them. Various extraction methods, such as Soxhlet, microwave-assisted extraction, and supercritical fluid extraction, are employed to extract polyphenols from plant biomass. Among the various extraction techniques, UAE’s advantage is that it can reduce extraction time and energy with eco-friendly green solvent including water and ethanol. Therefore, UAE is recognized as a suitable process for mass extraction of polyphenols by providing the advantages of effective mass transfer, reduced extraction temperature, use of green solvent, and decreased facility size. In conclusion, the UAE, which uses green solvents, is considered an appropriate alternative to existing extraction techniques in industrial scale extraction technology for the production of bioactive compounds.

Currently, there is a growing worldwide interest in discovering new, safe, and effective bioactive materials, such as antioxidant, anticancer, anti-allergic, and antimutagenic agents, from natural sources to minimize oxidative damage, allergic reactions, and mutagenesis to living cells. Efforts to use these natural functional materials for commercial purposes, such as medicine, cosmetics, and functional foods, have steadily been increasing. Therefore, the present process could be applied on a commercial scale in the production of bioactive materials from LEM, and the present results could form the basis for using LEM in further research for the potential discovery of novel natural bioactive materials from this by-product of algal biodiesel production. Taken together, these results suggest that LEM has an antioxidant ability and optimal extraction conditions of LEM will be useful for the development of food and pharmaceutical applications.

## Figures and Tables

**Table 1:** Independent variables and their coded and actual levels for central composite design (CCD).

Variables		Coded and Actual Levels				
		−2	−1	0	+1	+2
X_1_	Temperature (°C)	50	60	70	80	90
X_2_	Ethanol concentration (%)	21.8	40.0	58.3	76.5	94.8
X_3_	Extraction time (min)	15	30	45	60	75
X_4_	Ultrasonic power (W)	300	400	500	600	700

**Table 2:** Coded and experimental levels of the ultrasound-assisted extraction (UAE) operational parameters and experimental values for 27 experimental runs.

Run No.	X_1_	X_2_	X_3_	X_4_	TPC (mg GAE/g)	AA (%)
1	60 (−1)	40.0 (−1)	30 (−1)	400 (−1)	5.07	22.7
2	80 (+1)	40.0 (−1)	30 (−1)	400 (−1)	6.66	59.8
3	60 (−1)	76.5 (+1)	30 (−1)	400 (−1)	6.47	53.5
4	80 (+1)	76.5 (+1)	30 (−1)	400 (−1)	7.21	60.6
5	60 (−1)	40.0 (−1)	30 (−1)	400 (−1)	6.28	51.6
6	80 (+1)	40.0 (−1)	30 (−1)	400 (−1)	7.78	45.0
7	60 (−1)	76.5 (+1)	60 (+1)	400 (−1)	6.51	54.1
8	80 (+1)	76.5 (+1)	60 (+1)	600 (+1)	6.05	72.3
9	60 (−1)	40.0 (−1)	60 (+1)	600 (+1)	5.72	42.4
10	80 (+1)	40.0 (−1)	30 (−1)	600 (+1)	7.64	65.0
11	60 (−1)	76.5 (+1)	30 (−1)	600 (+1)	6.88	57.0
12	80 (+1)	76.5 (+1)	30 (−1)	600 (+1)	7.48	58.9
13	60 (−1)	40.0 (−1)	30 (−1)	600 (+1)	7.40	63.1
14	80 (+1)	40.0 (−1)	30 (−1)	600 (+1)	9.06	84.8
15	60 (−1)	76.5 (+1)	60 (+1)	600 (+1)	8.33	72.5
16	80 (+1)	76.5 (+1)	60 (+1)	500 (0)	9.41	72.3
17	50 (−2)	58.3 (0)	45 (0)	500 (0)	5.39	24.7
18	90(+2)	58.3 (0)	45 (0)	500 (0)	9.23	64.1
19	70 (0)	21.8 (−2)	45 (0)	500 (0)	5.10	48.7
20	70 (0)	94.8 (+2)	45 (0)	500 (0)	6.50	54.1
21	70 (0)	58.3 (0)	15 (−2)	500 (0)	5.26	25.2
22	70 (0)	58.3 (0)	75 (+2)	500 (0)	8.68	70.6
23	70 (0)	58.3 (0)	45 (0)	300 (−2)	8.68	70.6
24	70 (0)	58.3 (0)	45 (0)	700 (+2)	8.24	72.5
25	70 (0)	58.3 (0)	45 (0)	500 (0)	8.77	77.3
26	70 (0)	58.3 (0)	45 (0)	500 (0)	8.72	79.2
27	70 (0)	58.3 (0)	45 (0)	500 (0)	8.63	81.6

X_1_: Temperature; X_2_: Ethanol concentration; X_3_: Extraction time; X_4_: Ultrasonic power.

**Table 3:** Response variables and their fitted model equations.

Symbol	Response Variable	Quadratic Equation
Y_TPC_	Total phenolic compounds (mg GAE/g DW)	8.71 + 0.68X_1_ + 0.23X_2_ + 0.61X_3_ + 0.38X_4_ − 0.29X_1_X_2_ − 0.067X_1_X_3_ + 0.12X_1_X_4_ − 0.20X_2_X_3_ + 0.11X_2_X_4_ + 0.33X_3_X_4_ − 0.35X_1_^2^ − 0.73X_2_^2^ − 0.44X_3_^2^ − 0.064X_4_^2^
Y_AA_	DPPH (%)	76.03 + 7.52X_1_ + 3.23X_2_ + 7.78X_3_ + 4.18X_4_ − 2.99X_1_X_2_ − 2.23X_1_X_3_ − 0.61X_1_X_4_ − 0.84X_2_X_3_ − 3.50X_2_X_4_ + 2.69X_3_X_4_ − 7.13X_1_^2^ − 5.38X_2_^2^ − 6.26X_3_^2^ − 0.34X_4_^2^

X_1_: Temperature (°C); X_2_: Ethanol concentration (%); X_3_: Extraction time (min); X_4_: Untrasonic power (W).

**Table 4:** Analysis of variance (ANOVA) for effects of ultrasound-assisted extraction (UAE) process variables on extraction of TPC and AA using a quadratic model.

	TPC			AA		
	Sum of Squares	*F*-Value	*p-*Value	Sum of Squares	*F-*Value	*p-*Value
Model	3.046	6.105	0.002	447.240	4.997	0.004
X_1_	11.084	22.214	0.001	1359.015	15.184	0.002
X_2_	1.274	2.554	0.136	250.907	2.803	0.120
X_3_	8.797	17.630	0.001	1450.815	16.209	0.002
X_4_	3.383	6.779	0.023	418.335	4.674	0.052
X_1_X_2_	1.387	2.779	0.121	142.803	1.595	0.231
X_1_X_3_	0.072	0.143	0.712	79.210	0.885	0.365
X_1_X_4_	0.223	0.447	0.516	6.003	0.067	0.800
X_2_X_3_	0.628	1.259	0.284	11.223	0.125	0.729
X_2_X_4_	0.209	0.419	0.529	196.000	2.190	0.165
X_3_X_4_	1.736	3.479	0.087	115.563	1.291	0.278
X_1_^2^	2.634	5.278	0.040	1353.271	15.120	0.002
X_2_^2^	11.333	22.713	0.001	823.916	9.205	0.010
X_3_^2^	4.062	8.141	0.015	1072.260	11.980	0.005
X_4_^2^	0.087	0.174	0.684	29.558	0.330	0.576

X_1_: Temperature; X_2_: Ethanol concentration; X_3_: Extraction time; X_4_: Ultrasonic power.

**Table 5:** Experimental values and predicted values of response variables at optimum extraction conditions.

Response Variables	Optimum Extraction Conditions ^1^				Maximum Value	
	X_1_	X_2_	X_3_	X_4_	Predicted	Validation ^2^
TPC	74.4	55.4	59.6	700 W	10.2 mg GAE/g DW	9.8 mg GAE/g DW (±0.23)
AA					90.5%	91.8% (±0.50)
EGCG					0.12 mg/g DW	0.12 mg/g DW (±0.008)

^1^ X_1_: Temperature (°C); X_2_: Ethanol concentration (%); X_3_: Time; X_4_: Ultrasonic power (W). ^2^ Experimental results were expressed as average values ± standard deviation (*n* = 3).

## References

[1] Alliwell B. (1991). Reactive oxygen species in living systems: Source, biochemistry, and role in human disease. Am. J. Med..

[2] Li S., Chen G., Zhang G., Wu M., Wu S., Liu Q. (2014). Research progress of natural antioxidants in foods for the treatment of diseases. Food Sci. Hum. Well..

[3] Carolina S., Giorgio Z., Alberto M., Marco V., Gianni S., Arianna G., Luca M.N. (2018). Oxidative stress: Role of physical exercise and antioxidant nutraceuticals in adulthood and aging. Oncotarget.

[4] Lobo V., Patil A., Phatak A., Chandra N. (2010). Free radicals, antioxidants and functional foods: Impact on human health. Pharmacogn. Rev..

[5] Taghvaei M., Jafar S.M. (2015). Application and stability of natural antioxidants in edible oils in order to substitute synthetic additives. J. Food Sci. Technol..

[6] Kahl R. (1984). Synthetic antioxidants: Biochemical actions and interference with radiation, toxic compounds, chemical mutagens and chemical carcinogens. Toxicology.

[7] Lindenschmidt R.C., Tryka A.F., Goad M.E., Witschi H.P. (1986). The effects of dietary butylated hydroxytoluene on liver and colon tumor development in mice. Toxicology.

[8] Barlow S.M. (1990). Toxicological aspects of antioxidants used as food additives. Food Antioxidants.

[9] Kahl R., Kappus H. (1993). Toxicology of the synthetic antioxidants BHA and BHT in comparison with the natural antioxidant vitamin E. Eur. Food Res. Technol..

[10] Metcalf R.L. (1987). Plant volatiles as insect attractants. Crit. Rev. Plant Sci..

[11] Ralston L., Subramanian S., Matsuno M., Yu O. (2005). Partial reconstruction of flavonoid and isoflavonoid biosynthesis in yeast using soybean type I and type II chalcone isomerases. Plant Physiol..

[12] Pardo J.M., Hasegawa P., Bressan R.A., Narasimhan M.L. (2003). In defense against pathogens: Both plant sentinels and foot soldiers need to know the enemy. Plant Physiol..

[13] Kazeem M.I., Akanji M.A., Hafizur R.M., Choudhary M.I. (2012). Antiglycation, Antioxidant and Toxicological Potential of Polyphenol Extracts of Alligator Pepper, Ginger and Nutmeg from Nigeria. Asian Pac. J. Trop. Biomed..

[14] Konaté K., Hilou A., Mavoungou J.F., Lepengué A.N., Souza A., Barro N., Datté J.Y., M’Batchi B., Nacoulma O.G. (2012). Antimicrobial Activity of Polyphenol-Rich Fractions from *Sida alba* L. (Malvaceae) against Cotrimoxazol-Resistant Bacteria Strains. Ann. Clin. Microbiol. Antimicrob..

[15] Valentão P., Fernandes E., Carvalho F., Andrade P.B., Seabra R.M., Bastos M.L. (2002). Studies on the antioxidant activity of *Lippia citriodora* infusion: Scavenging effect on superoxide radical, hydroxyl radical and hypochlorous acid. Biol. Pharm. Bull..

[16] Choi H.R., Choi J.S., Han Y.N., Bae S.J., Chung H.Y. (2003). Peroxynitrite scavenging activity of herb extracts. Phytother. Res..

[17] Bouterfas K., Mehdadi Z., Benmansour D., Khaled M.B., Bouterfas M., Latreche A. (2014). Optimization of Extraction Conditions of Some Phenolic Compounds from White Horehound (*Marrubium vulgare* L.) Leaves. Int. J. Org. Chem..

[18] Herrero M., Sánchez A.P., Alejandro C., Elena I. (2015). Plants, seaweeds, microalgae and food by-products as natural sources of functional ingredients obtained using pressurized liquid extraction and supercritical fluid extraction. TrAC Trend Anal. Chem..

[19] Goiris K., Muylaert K., Ilse F., Foubert I., Brabanter J., Cooman L.C. (2012). Antioxidant potential of microalgae in relation to their phenolic and carotenoid content. J. Appl. Phycol..

[20] Singh B., Singh N., Thakur S., Kaur A. (2017). Ultrasound assisted extraction of polyphenols and their distribution in whole mung bean, hull and cotyledon. J. Food Sci. Technol..

[21] Da Porto C., Porretto E., Decorti D. (2013). Ultrasonics Sonochemistry Comparison of ultrasound-assisted extraction with conventional extraction methods of oil and polyphenols from grape (*Vitis vinifera* L.) seeds. Ultrason. Sonochem..

[22] Dent M., Dragović-Uzelac V., Elez G.I., Bosiljkov T., Ježek D., Brnčić M. (2015). Comparison of conventional and ultrasound-assisted extraction techniques on mass fraction of phenolic compounds from Sage (*Salvia officinalis* L.). Chem. Biochem. Eng..

[23] Nelly M.T., Teresa A.T., Hugo E.A., Angeles S.C., Neith P. (2017). Ultrasound assisted extraction for the recovery of phenolic compounds from vegetable sources. Agronomy.

[24] Fang X., Wang J., Wang Y., Li X., Zhou H., Zhu L. (2014). Optimization of ultrasonic-assisted extraction of wedelolactone and antioxidant polyphenols from *Eclipta prostrate* L using response surface methodology. Sep. Purif. Technol..

[25] Gajic S., Savic I., Boskov I., Žerajić S., Markovic I., Gajic D. (2019). Optimization of Ultrasound-Assisted Extraction of Phenolic Compounds from Black Locust (*Robiniae Pseudoacaciae*) Flowers and Comparison with Conventional Methods. Antioxidants.

[26] Kong F., Yu S., Feng Z., Wu X. (2015). Optimization of ultrasonic-assisted extraction of antioxidant compounds from Guava (*Psidium guajava* L.) leaves using response surface methodology. Pharmacogn. Mag..

[27] Tan M.C., Tan C.P., Ho C.W. (2013). Effects of extraction solvent system, time and temperature on total phenolic content of henna (*Lawsonia inermis*) stems. Int. Food Res..

[28] Liu Y., Hu Q. (2015). Optimization of extraction process for total phenols from adlay. Eur. J. Food Sci. Technol..

[29] Lv L., Wei L., Chen D., Liu J., Lin S., Ye H., Yuan Y. (2018). Optimization of ultrasound-assisted extraction of polyphenols from maize filaments by response surface methodology and its identification. J. Appl. Bot. Food Qual..

[30] Nur F.A., Siti S.A.G., Nor F.M.M. (2017). Optimization of phenolics and flavonoids extraction conditions of *Curcuma zedoaria* leaves using response surface methodology. Chem. Cent. J..

[31] Lapornik B., Prosek M., Wondra A.G. (2005). Comparison of Extracts Prepared from Plant By-Products Using Different Solvents and Extraction Time. J. Food Eng..

[32] Snitsarev V., Young M.N., Miller R.M.S., Rotella D.P. (2013). The Spectral Properties of (−)-Epigallocatechin 3-O-Gallate (EGCG) Fluorescence in Different Solvents: Dependence on Solvent Polarity. PLOS ONE.

[33] Fu H.Y., Shieh D.E., Ho C.T. (2002). Antioxidant and free radical scavenging activities of edible mushrooms. J. Food Lipids.

[34] Maran J.P., Nivetha C.V., Al-Dhabi B.P., Ponmurugan K., Manoj J.J. (2016). Modeling of polysaccharide extraction from *Gossypium arboreum* L. seed using central composite rotatable design. Int. J. Biol. Macromol..

